# Effectiveness of interventions for people bereaved through suicide: a systematic review of controlled studies of grief, psychosocial and suicide-related outcomes

**DOI:** 10.1186/s12888-019-2020-z

**Published:** 2019-01-30

**Authors:** Karl Andriessen, Karolina Krysinska, Nicole T. M. Hill, Lennart Reifels, Jo Robinson, Nicola Reavley, Jane Pirkis

**Affiliations:** 1 Centre for Mental Health, Melbourne School of Population and Global Health, The University of Melbourne, 207 Bouverie St, Melbourne, VIC 3010 Australia; 2 School of Psychiatry, University of New South Wales, Hospital Rd, Randwick, NSW 2031 Australia; 3 Centre for Primary Health Care and Equity, University of New South Wales, Sydney, NSW 2052 Australia; 4 Orygen, The National Centre of Excellence in Youth Mental Health, The University of Melbourne, 35 Poplar Road, Parkville, VIC 3052 Australia

**Keywords:** Bereavement, Effectiveness, Grief, Interventions, Postvention, Suicide, Systematic review

## Abstract

**Background:**

Suicide bereavement is a risk factor for adverse outcomes related to grief, social functioning, mental health and suicidal behaviour. Consequently, suicide bereavement support (i.e., postvention) has been identified as an important suicide prevention strategy. However, little is known about its effectiveness. To redress this gap, this review aimed to assess the evidence of effectiveness of interventions for people bereaved by suicide, and appraise the quality of the research in this field.

**Methods:**

We conducted a systematic review according to PRISMA guidelines. Searches of peer-reviewed literature in Medline, PsycINFO, Embase and EBM Reviews identified 12 papers reporting on 11 relevant studies conducted between 1984 and 2018.

**Results:**

Across studies, there was a wide variety of intervention modalities, study populations, control groups, and grief, psychosocial and suicide-related outcome measures. Overall, the quality of studies was weak. While there was some evidence of the effectiveness of interventions for uncomplicated grief, evidence of the effectiveness of complicated grief interventions was lacking. Based on this scant evidence, interventions which seem to show promise include supportive, therapeutic and educational approaches, involve the social environment of the bereaved, and comprise a series of sessions led by trained facilitators.

**Conclusions:**

There is a clear need for additional methodologically sound studies in this area. Specifically, selection procedures, sample sizes, randomization, and the use of appropriate measures are crucial. As people bereaved by suicide are at-risk of adverse grief, mental ill-health and suicidal behaviour, further research across the life-span is essential to prevent grief and mental health ramifications.

## Background

Suicide constitutes a major public health problem with more than 800,000 people dying by suicide globally each year [1]. The societal toll of suicide goes well beyond the human loss. Whereas the act of suicide ends the pain of one, it is a major disruptive and psychosocial stressor for others who are bereaved [2]. A suicide death can affect a substantial number of people. For example, Berman [3] found that one suicide can affect between five nuclear family members and 80 relatives, friends and acquaintances. A recent meta-analysis of population-based studies found that approximately one in 20 people (4.3%) have experienced a suicide in one year, and one in five (21.8%) have done so during their lifetime [4].

Grief is understood as the primarily emotional (affective) and natural reaction to the loss of a significant other [5]. It encompasses diverse psychological (emotional, cognitive), physical, and behavioural responses to the death. Acute grief reactions include sadness, crying, yearning, guilt and anger [5]. The course and duration of the grief process after a suicide death appear to be similar to grief processes following deaths by other causes [6, 7]. Nonetheless, people bereaved by suicide may experience more shock or trauma related to the unexpected or violent nature of the death compared to other forms of bereavement [7]. They may also experience more feelings of abandonment, rejection, shame, struggles with meaning-making and ‘why’-questions, and less social support [8, 9].

Suicide bereavement is also a risk factor for adverse outcomes related to complicated grief [10]. While consensus is emerging about the diagnostic criteria and the name of the syndrome (Prolonged Grief Disorder will be included in ICD-11), it is expressed through persisting characteristics of acute grief, and is more likely to occur after a sudden or violent death [11]. While there may be similarities between some clinical characteristics of complicated grief, depression and posttraumatic stress disorder (PTSD), there are a number of specific symptoms (i.e., maladaptive reactions) of complicated grief such as intense longing for the deceased, ruminative thoughts or images about the deceased, intense feelings of anger and guilt, avoidance of situations, people and places that remind of the deceased, and difficulty finding meaning in life [12, 13].

Compared with the general population, people bereaved by suicide have a higher risk of suicidal behaviour, and psychiatric problems such as depression, anxiety, post-traumatic stress disorder, and substance abuse [8, 14]. This is particularly the case for those who have a personal or family history of mental health and suicidal behaviour [15, 16]. There is also growing evidence of increased physical disorders among people bereaved by suicide, possibly related to increased unhealthy lifestyles (e.g., poor diet, smoking) after the bereavement [16–18].

More than four decades ago, Shneidman [19] identified provision of adequate suicide bereavement support (i.e., postvention) as a major public and mental health challenge. Currently there seems to be a tension between the need for psychosocial and professional support reported by people bereaved by suicide [20–23] and what is known about its effectiveness [24–26]. At the same time, postvention has become available in an increasing number of countries [27], and has been recognized as an important suicide prevention strategy [1]. There has also been an increase in suicide bereavement research [28–30]. However, most of the research has been focused on the experiences of those who have been bereaved and the characteristics of suicide bereavement, whereas the effectiveness of postvention in terms of its impact on the grief process and mental health of bereaved individuals remains unclear [8].

To date three systematic reviews of interventions for people bereaved by suicide have been published [24–26]. McDaid et al. [24] identified eight controlled studies of grief and psychosocial interventions, delivered mostly in a family or group context. Six interventions showed some evidence of effectiveness on at least one outcome measure, such as reduced anxiety or depression, and less maladaptive grief reactions. Szumilas and Kutcher [25] reviewed sixteen postvention programs, including school-based, community-based and family-focused interventions. Although some of the reviewed interventions had positive impact on mental health and grief outcomes, the review found no evidence of reduced incidence of non-fatal or fatal suicidal behaviour related to any of the programs. Linde et al. [26] reported on seven group and individual intervention studies specifically in the context of grief-related outcomes, encompassing complicated grief, uncomplicated grief and suicide-specific aspects of grief, such as guilt, responsibility and rejection. Five studies demonstrated a decrease of intensity of grief on at least one measure.

The three systematic reviews conducted to-date found some evidence of effectiveness of postvention interventions [24–26]. They also pointed out the limitations of the field, namely the paucity of intervention research in postvention, the diversity of methodologies used, and the general poor quality of the relevant studies. However, the systematic reviews used different inclusion and exclusion criteria and reported on different outcomes, ranging from a variety of grief and mental health outcomes [24] to grief-specific outcomes [26]. In addition, although McDaid et al. [24] focused on controlled studies only, including randomized controlled trials (RCTs), Szumilas and Kutcher [25] applied a wide perspective and included both controlled and uncontrolled studies, and a recent review by Linde et al. [26] also included an uncontrolled study. The differences between these reviews (i.e., broad criteria regarding study design or narrow criteria regarding outcomes) make it difficult to draw conclusions about the effectiveness of interventions for people bereaved by suicide.

To redress this gap in the literature and postvention practice, this review aimed to establish the effectiveness of suicide bereavement interventions with regard to grief, psychosocial (related to mental health and psychological functioning) and suicide-related outcomes using data from controlled studies only. The review was designed to (1) uncover the research findings regarding the effectiveness of interventions on grief, psychosocial and suicide-related outcomes, (2) assess the quality of the included studies, and (3) consider the implications for practice and further research. The findings of this review will provide crucial information for service providers, both professional and peer support-based, and policy makers, as well as for the bereaved by suicide in need of effective support.

## Method

The review was conducted following the PRISMA guidelines [31], with systematic searches of the following databases: Medline, PsycINFO, Embase, and EBM Reviews, accessed through Ovid. Medline was searched with a combination of MeSH and text words: (bereavement/ OR bereavement.mp OR grief/ OR grief.mp OR mourning.mp) AND (family/ OR relative.mp OR spouse.mp OR parent.mp OR sibling.mp OR grandparent.mp OR widow.mp OR child.mp OR acquaintance.mp OR friends/ OR friends.mp OR students/ OR student.mp OR schools/ OR school.mp OR survivor.mp OR suicide survivor.mp) AND (counseling/ OR counseling.mp OR intervention.mp OR postvention.mp OR psychotherapy/ OR psychotherapy.mp OR psychoeducation.mp OR therapy.mp OR treatment.mp OR support.mp OR support group.mp OR self-help groups/ OR social media/ OR social media.mp OR internet/ OR internet.mp OR online.mp) AND (suicide/ OR suicide.mp OR suicide cluster.mp). We have used the same search string in the other databases using subject headings and keywords.

**Fig. 1:**
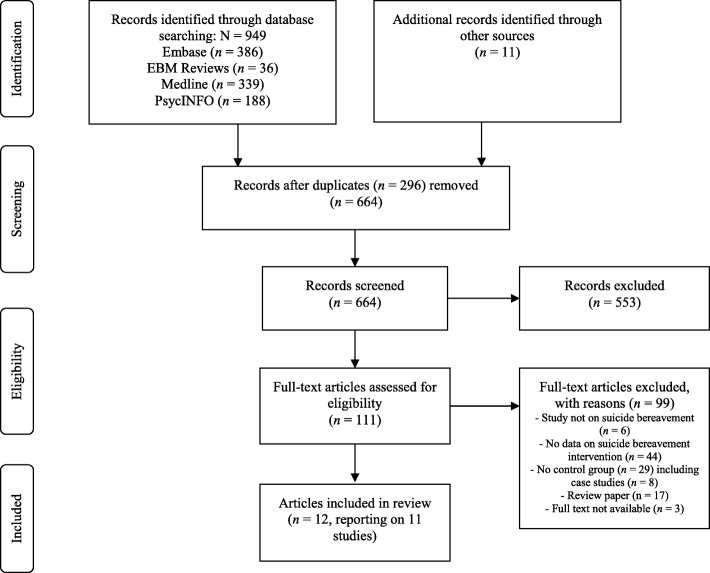
PRISMA Flow Diagram

The search was undertaken in August 2018 and was not limited by language or date of publication. Three researchers (KA, KK, NH) independently assessed titles and abstracts for eligibility. Any disagreement was resolved through discussion. Potentially relevant studies were examined against the inclusion/exclusion criteria. The references of retrieved papers and existing reviews were hand searched to identify additional studies. Figure 1 presents the search and selection process.

### Inclusion and exclusion criteria

The following inclusion criteria were used: (1) study population consists of people bereaved by suicide, (2) study provides empirical data on grief, mental health and/or suicide-related (i.e., suicidal ideation and/or (non-)fatal suicidal behavior) outcomes, (3) study involves a controlled intervention, and (4) study is published as a paper in a peer-reviewed journal. The review excluded: (1) studies not providing data specifically on people bereaved by suicide, (2) studies not providing data on grief, mental health and/or suicide-related outcomes, (3) studies without a control group, (4) case studies, and (5) review papers.

### Data extraction

Three researchers (KA, KK, NH) independently extracted the following data from the selected studies: author, year and location (country), study design, eligibility criteria, sample size, participants’ age and sex distribution, participants’ time since the bereavement and relationship to the deceased, type (individual, family, group), characteristics and duration of the intervention, outcome measures, names of the instruments used, and main results of the study. Any disagreement was resolved through discussion.

### Quality assessment

The methodological quality of the included studies was assessed with the Quality Assessment Tool for Quantitative Studies [32]. No qualitative study met the inclusion criteria. The instrument comprises six components (selection bias, study design, confounders, blinding, data collection methods, and withdrawals and dropouts) to be scored as weak, moderate or strong. The total rating of a study was ‘strong’ if none of its components were rated ‘weak’. A study was rated ‘moderate’ if only one of its components was rated ‘weak’, and received a total rating of ‘weak’ if two or more of its components were rated as ‘weak’ [32]. In addition, the instrument assesses the integrity of the intervention and analyses (e.g., analysis by intention to treat status). Two researchers (KK, NH) independently assessed the quality of the included studies. Discussion with a third researcher (KA) and the wider team resolved any disagreement.

## Results

### Study characteristics

The systematic search identified 12 papers meeting the inclusion criteria. These reported on 11 studies, published between 1984 and 2018 (Table 1). Eight studies were conducted in the USA [33–40]. The other three were conducted in the Netherlands [41, 42], Australia [43], and Belgium [44]. Five studies had a passive control group (i.e., no intervention) [36, 38, 39, 43, 44]. Six studies included an active control group [33–35, 37, 40] or a treatment-as-usual control condition [41, 42].

**Table 1:** Effectiveness of suicide bereavement interventions: Summary of studies

Author (year)/Location	Eligibility criteria	Sample size	Age (M, SD, range)	Male/female	Time since bereavement/Relationship to the deceased	Type of intervention/Setting	Characteristics of intervention	Duration/Frequency of contact	Outcome/Instrument/Timepoints	Main results
Battle (1984) USA [33]	Intervention: suicide loss, help-seeking from a crisis centre Control: i) Non-help-seeking suicide survivors (non-group control group), ii) patients from the centre (patient control group).	Intervention *N* = 36 Non-group bereaved control group *N* = 13 Patients control group *N* = 31	Range 14–66 years *M* = 38 Control groups: not reported	22% /78% Control groups: not reported	8 days – 11 years M = 2 months	Group Suicide prevention/crisis intervention service	Support group with educational component non-help seeking survivors, and patients Combination of non-directive and directive leadership Facilitated by clinicians	1.5 h weekly sessions in first 4 months, followed by 2-weekly 1.5 h sessions 69% attend less than 10 sessions 97% attends max 14 sessions.	Psychosocial functioning incl. Problems, feelings, goals (self-constructed questions) Pre/post intervention	Participants are more emotional (more distress, pain, happiness, pleasure) than non-group controls); suicidal pain 50% vs 0%; perfect happiness: 67% vs 1%; more solutions to their problems than controls. Control patients also experienced strong emotions. Psychodynamic insights in the survivors and the suicides. No *p* values reported.
Constantino & Bricker (1996) USA [34]	Widows whose spouse died by suicide	Intervention *N* = 16 Control *N* = 16	M = 43	Majority is female More men in intervention group than in control	Not reported	Group Setting not reported	Bereavement Group Postvention (BGP), i.e. group psycho therapy vs Social Group Postvention (SGP), e.g., socialization, recreation Directive vs non-directive leadership Facilitated by trained mental health nurses	1.5 h weekly sessions, 8 weeks	Depression (BDI) Distress (BSI) Social adjustment (SAS) Grief (GEI) Pre/post intervention	Reduction in depression and distress in both BGP and SGP groups (both *p* < .05). Improvement in social adjustment in SGP group only (*p* = .003). Grief aspects: despair, rumination and depersonalization decreased in both BGP and SGP groups (all *p* < .05); anger/hostility and guilt decreased in BGP only (*p* < .05).
Constantino et al. (2001) USA [35]	Widows whose spouse died by suicide Aged 18+ English speaking	*N* = 47 (Originally *N* = 60, 30 + 30)	Range 24–70 yrs.	Male/female 10/37, 21%/78%	1–27 months M = 10.91, *SD* = 8.65 40% less than 6 months Widows	Group Setting not reported	Bereavement Group Postvention (BGP), i.e. group psycho therapy vs Social Group Postvention (SGP), e.g., socialization, recreation Facilitated by trained mental health nurses	1.5 h weekly sessions, 8 sessions	Depression (BDI) Distress (BSI) Grief (GEI) Social adjustment (SAS) Pre/post intervention, 6-month, 12-month follow-up	N.s. differences between BGP and SGP groups on outcome variables at four timepoints. Analysis of both groups BGP and SGP combined: significant decrease in depression (*p* =. 0001), total distress (*p* = .0001), grief symptoms (all *p* < .05), except for anger/hostility and social isolation, and increase in total social adjustment (*p* = .0001) over the four time points.
De Groot et al. (2007) The Netherlands [41]	First-degree relatives or spouses bereaved by suicide Aged 15+	Intervention *N* = 68, 39 families Control *N* = 54, 31 families	Intervention M = 43, *SD* = 13.7 Control M = 43, *SD* = 13.5	Intervention Male/female 28/40, 41%/59% Control Male/female 12/42, 22%/78%	3–6 months after suicide Spouse (31%) Parent (31%) Child (16%) In-laws (4%)	Group / family Participant’s home	Family-based cognitive behaviour counselling programme vs treatment as usual Facilitated by trained psychiatric nurses	2 h, 2- -3 weekly sessions, 4 sessions	Grief (ITG, TRGR2L) Depression (CES-D) Suicidal ideation (PSI) Guilt and self-blame (self-constructed questions) Baseline, 13-month follow-up	N.s. effect of intervention on complicated grief, depression, and suicidal ideation. A trend towards feeling less being to blame (*p* = .01) and fewer maladaptive grief reactions (*p* = .056).
De Groot et al. (2010) The Netherlands [42]	First-degree relatives or spouses bereaved by suicide Suicide < 8 weeks Aged 15+	Total *N* = 122 Intervention *N* = 68, 39 families Control *N* = 54, 31 families	Intervention M = 43, *SD* = 13.7 Control M = 43, *SD* = 13.5	Intervention Male/female 28/40, 41%/59% Control Male/female 12/42, 22%/78%	3–6 months after suicide Spouse (31%) Parent (31%) Child (16%) In-laws (4%)	Group / family Participant’s home	Family-based cognitive behaviour counselling programme vs treatment as usual Facilitated by trained psychiatric nurses	2 h, 2–3 weekly sessions, 4 sessions	Grief (ITG, TRGR2L) Depression (CES-D) Suicidal ideation (PSI) Guilt and self-blame (self-constructed questions) Clinical assessment (SCAN2.1) Baseline, 13-month follow-up	Participant with suicidal ideation compared with non-ideators: N.s. decrease of complicated grief in suicide ideators Reduction in maladaptive grief reactions (*p* = .03) and risk of suicidal ideation (*p* = .03) among ideators.
Farberow (1992) USA [36]	Loss by suicide Aged 18+	Intervention *N* = 60 Control *N* = 22	Range 18–60+	Intervention Male/female 18/42, 30%/70% Control 5/17, 23%/77%	Less than 3 to 24+ months 77% between 6 and 8 months after death Sibling (35%) Child (23%) Parent (20%) Spouse (13%) Sweathearts and other	Group Suicide prevention centre	Bereavement group with therapeutic and educational aspects vs refusers, dropouts Quasi-experimental design Facilitated by mental health professional and trained peer	1.5 h weekly sessions, 8 sessions, followed by open monthly sessions	Health, impact of loss, coping, major changes, feelings (self-constructed questions) After suicide (T1), pre/post intervention (T2/T3)	Intervention group: decreased scores from T1 to T2 to T3. Control group: decreased scores from T1 to T2; only in anxiety for T2 to T3. Intervention group at T3 significant higher on depression and puzzlement compared to Control group. No* p* values reported.
Hazell & Lewin (1993) Australia [43]	Students selected by school staff on basis of close friendship with deceased student	Intervention *N* = 63 Control *N* = 63	School A: M = 15.1 School B: M = 14.4	Not reported	Within 7 days after suicide Fellow students of same school	Group School setting	Group counselling and information vs no counselling Facilitated by child psychiatrist or trainee psychiatrist with assistance of senior school staff	1.5 h session One session	Behaviour (YSR) Risk behaviour (RBQ) Suicidal ideation/behaviour Drug/alcohol use 8 months after suicide	N.s. differences between intervention and control group on internalizing**,** externalizing, depression, risk behaviour, suicidal ideation/behaviour or drug/alcohol use.
Kovac & Range (2000) USA [37]	Undergraduate students who had a close person die by suicide in the past 2 years and were upset by the death	Total *N* = 42 Intervention *N* = 20 Control *N* = 22 *N* = 30 completed follow-up tests	Range 18–46 M = 23.98 *SD* = 7.34 Intervention M = 23.16, *SD* = 6.99 Control M = 25, *SD* = 7.98	Male/female 9/33, 21%/79% Intervention 5/14, 26%/74% Control 3/18, 14%/86%	Intervention M = 13.26 months, *SD* = 9.32 Control M = 11.95, *SD* = 6.54 Not reported	Individual Experimental/laboratory setting	Writing task: profound, death-related writing vs trivial writing Facilitated by researchers	15 min sessions, 4 sessions over 2 weeks	Grief (GRQ, GEQ) Impact of grief (IES) Essay evaluation form Experiment follow-up form Pre/post-test (6 weeks)	Reduction in impact of grief (*p* < .05), and general GRQ grief levels (*p* < .05) in intervention and control group. Suicide-specific grief GEQ more reduced in intervention than control group (*p* < .05). No difference in self-reported health visits between groups.
Pfeffer et al. (2002) USA [38]	Families where child’s parent or sibling died by suicide Children aged 6–15 No psychiatric disorders	Total *N* = 75 children, 52 families Intervention *N* = 39, 27 fam Control *N* = 36, 25 fam	Intervention M = 9.6, *SD* = 2.9 Control M = 11.4, *SD* = 3.5	Male/female Intervention 16/23, 41%/59% Control 12/24, 33%/67%	Within a year after death Siblings (11/39), children (28/39) and parents	Group / family Clinical setting	Manual based bereavement group intervention for children grouped by age Psycho-educational, support group for parents No treatment control Facilitated by trained psychologists	1.5 h weekly sessions 10 sessions	Children: Posttraumatic stress symptoms (CPTSRI) Depression (CDI) Anxiety (RCMAS) Social adjustment (SAICA) Parents: depression (BDI) Pre/post intervention (12 weeks)	Children: Significantly greater reduction in anxiety and depressive symptoms in intervention vs. control group (*p* ≤ .01). N.s. differences in posttraumatic stress or social adjustment. Parents: N.s. differences in depression between groups.
Sandor et al. (1994) USA [39]	Members of a church-related youth group	Intervention *N* = 15 Control *n* = 19	Intervention Range 14–17, M = 15.73 Control range 14–18, M = 16.37	Intervention Male/female 5/15, 33%/67%r Control Male/female 6/13, 32%/68%	A few days after the death Relationship: peer group	Group Church youth group	Supportive community intervention; Survivor Group (SG) vs Comparison Group (CG; no intervention) Quasi experimental design Facilitated by church youth group leaders	3 meetings: 2 h open session with youth and parents, after two days one closed psycho-educational session with youth, a day later a memorial service in church	Problem solving (APSA) Self-perception (HSP) Self-efficacy (SES) Baseline (T1), 2 months after suicide (T2), 2-month follow-up (T3)	Greater self-efficacy at T2 and T3 compared to T1 in SG vs. CG group (*p* < .01). Greater social acceptance and job competence at T2 in SG vs. CG (*p* < .05), but not at T3. SG vs CG group not compared on problem-solving appraisal, scholastic competence, and global self-worth.
Wittouck et al. (2014) Belgium [44]	Suicide of a significant other 3–24 months before participation Aged 18+ Dutch speaking	Intervention *N* = 47 Control *N* = 36	Intervention M = 49.3, *SD* = 13.8 Control M = 47.6, *SD* = 12.8	Intervention male/female 9/38, 19%/81% Control 11/25, 31%/69%	Intervention 9.8 months, *SD* = 5.7 Control M = 12.4, *SD* = 6.3 Deceased: child (*n* = 20; 42%), partner (*n* = 12, 25%), parent (*n* = 1, 2%), sibling (*n* = 8, 17%), other (*n* = 6, 13%)	Group / family Participant’s home	Cognitive-behavioral therapy-based psycho-educational intervention vs no treatment Facilitated by clinical psychologist	2 h sessions, 4 sessions Frequency not reported	Depression BDI-II) Hopelessness (BHS) Grief (CGQ, ITG) Coping (UCL) Baseline, 8 (months	N.s. decrease in depression, hopelessness and grief in intervention vs. control group. Decrease in intensity of grief, depression, passive coping style, social support seeking and behavioural expression of (negative) feelings in intervention group only (all *p* < .05).
Zisook et al. (2018) USA [40]	People bereaved by suicide SB), accident, homicide (A/H), and natural causes (NC) with ≥30 ITG score Exclusion: Being suicidal, other psychiatric disorders except depression, other treatments	Total *N* = 395 SB *N* = 58 A/H *N* = 74 NC *N* = 263 SB Medication *N* = 14 Placebo *N* = 17 CGT + Medication *N* = 17 CGT + Placebo *N* = 13	Range 18–95 SB M = 47.2, *SD* = 14.1 A/H M = 51.6, *SD* = 14.8 NC *N* = 54.6, *SD* = 14.2	Male/female SB 10/48, 17%/82% A/H 18/56, 24%/76% NC 59/204, 22%/78%	Time since death SB M = 3.9 yrs. *SD* = 4.6 A/H M = 6.6 *SD* = 9.1 NC 4.3 *SD* = 7.1 SB deceased is a partner 18 (31%), parent 7 (12%), child 19 (33%), other 14 (24%)	Individual Clinical setting	Manual-based structured Complicated Grief Therapy (CGT) Therapists, including social workers, psychiatrists, psychologists Antidepressant medication (citalopram)with individual follow-up Facilitated by Trained researcher	CGT: 16 sessions over 20 weeks Medication: 12-week with 2–4 weekly visits until week 20	Grief (CG-CGI-I, GRAQ, ICG, SCI-CG, TBAQ) Suicidal ideation (C-SSRS-R) Work/social adjustment (WSAS)	Lower improvement on clinician-rated CG-CGI-I in SB vs. A/H and NC groups (*p* < 0.5). N.s. differences on other measures of grief, suicidal ideation or work/social adjustment between SB, A/H and NC groups. Low rates of post treatment active suicidal ideation in SB, A/H and NC groups.

APSA: Adolescent Problem Solving Appraisal [60]; BDI: Beck Depression Inventory [48]; BHS: Beck Hopelessness Scale [61]; BSI: Brief Symptom Inventory [50]; CDI: Children’s Depression Inventory [62]; CES-D: Center for Epidemiological Studies Depression Scale [49]; CG-CGI-I: Complicated Grief Clinical Global Impressions Scale – Improvement [63]; CPTSRI: Childhood Posttraumatic Stress Reaction Index [64]; C-SSRS-r: Columbia Suicide Severity Rating Scale – Revised [52]; GCQ: Grief Cognitions Questionnaire [46]; GEI: Grief Experience Inventory [47]; GEQ: Grief Experience Questionnaire [65]; GRAQ: Grief-Related Avoidance Questionnaire [66]; GRQ: Grief Recovery Questions [57]; IES: Impact of Event Scale [67]; ITG: Inventory of Traumatic Grief [68]; PSI: Paykel’s Suicidality Items [69]; RBQ: Risk Behavior Questionnaire [70]; RCMAS: Revised Children’s Manifest Anxiety Scale [71]; SAICA: Social Adjustment Inventory for Children and Adolescents [72]; SAS: Social Adjustment Scale [51]; SCAN 2.1: Schedules for Clinical Assessment in Neuropsychiatry [73]; SCI-CG: Structured Clinical Interview for Complicated Grief [74]; SES: Self Efficacy Scale [75]; SPP: Self-Perception Profile for Adolescents [76]; TBQ: Typical Beliefs Questionnaire [77]; TRGR2L: Traumatic Grief Evaluation of Response to Loss [45]; UCL: Utrecht Coping List [78]; WSAS: Work and Social Adjustment Scale [79]; YSR: Youth Self Report Child Behavior Checklist [80]

Most studies involved interventions that targeted adult populations, but three tested interventions aimed at children or adolescents [38, 39, 43]. A few studies included participants aged 65+ (e.g., Zisook et al. [40]), but no study specifically involved interventions targeting older adults. The percentage of females in the intervention samples ranged from 59 to 82%. Studies varied in terms of the types of participants targeted. Some focused on those with specific relationships with the deceased, such as parents [38] or spouses [35], and some had a broader focus on ‘significant others’ (e.g., Wittouck et al. [44]). The time since the bereavement varied across studies, with some reporting a few days [43] and others several years (Zisook et al. [40] report *M* = 3.9 years in the suicide bereaved sample). However, about half of the studies reported either a time range or a mean within or just over one year since the bereavement [35, 37, 38, 41, 42, 44].

Six studies tested the effectiveness of a group intervention [33–36, 39], including a group/school-based intervention [43]. Three studies evaluated family-oriented interventions [38, 41, 42, 44], and two examined individual interventions [37, 40]. The group, family and individual interventions applied supportive, psychotherapeutic and psycho-educational approaches, involving interactions and exchange with peers and/or professionals. One intervention was based on an individual writing task but also involved interaction with the researchers [37]. The duration of the intervention varied from one session in one study [43] to a series of 16 therapeutic sessions delivered over 20 weeks in another [40]. Several studies involved manualised interventions [37, 38, 40–42]. As presented in Table 1, studies also varied in terms of the outcomes they assessed, and how they assessed them. Some studies assessed grief, using measures like the Inventory of Traumatic Grief [45], the Grief Cognitions Questionnaire [46], and the Grief Experience Inventory [47]. Other studies assessed mental health outcomes like levels of depressive symptoms (Beck Depression Inventory [48], Centre for Epidemiological Studies Depression Scale [49]), psychological distress (Brief Symptom Inventory [50]) or social adjustment (Social Adjustment Scale [51]). Still other studies assessed suicide-related outcomes, using measures like the Columbia Suicide Severity Rating Scale [52].

### Quality assessment

Table 2 details the methodological quality of the studies according to the six components of the Quality Assessment Tool for Quantitative Studies [32]. The overall study quality was weak: nine studies received a total rating of ‘weak’; the other two [37, 38] were rated ‘moderate’. Looking at the rating in detail, three studies [34, 41, 42, 44] were rated ‘strong’ on four components, and four studies [35, 38–40] were rated ‘strong’ on three components. Across studies, selection bias, blinding, and withdrawals and dropouts, were the weakest components. Seven studies used a randomized design; however, only one applied an intention-to-treat analysis [41, 42]. Also, whilst studies applied valid and reliable measures, it is unknown if studies controlled for effects of other treatments (e.g., by a family doctor) which participants might have been receiving.

**Table 2:** Quality assessment

Quality Criteria	Battle (1984) USA [33]	Constantino & Bricker (1996) USA [34]	Constantino et al. (2001) USA [35]	De Groot et al. (2007; 2010) The Netherlands [41, 42]	Farberow (1992) USA [36]	Hazell & Lewin (1993) Australia [43]	Kovac & Range (2000) USA [37]	Pfeffer et al. (2002) USA [38]	Sandor et al. (1994) USA [39]	Wittouck et al. (2014) Belgium [44]	Zisook et al. (2018) USA [40]
A. Selection bias											
Representativeness	Not likely	Not likely	Not likely	Somewhat likely	Somewhat likely	Somewhat likely	Somewhat likely	Somewhat likely	Can’t tell	Not likely	Not likely
Percentage agreed	Can’t tell	80–100%	Can’t tell	< 60%	Can’t tell	60–79%	60–79%	60–79%	Can’t tell	80–100%	Can’t tell
Rating	Weak	Weak	Weak	Weak	Moderate	Moderate	Moderate	Moderate	Weak	Weak	Weak
B. Study design											
Study design type	Other: 3 groups comparison	RCT	RCT	RCT	Cohort analytic	Case-control	RCT	RCT	Cohort analytic	RCT	RCT
Described as randomized?	No	Yes	Yes	Yes	No	No	Yes	Yes	No	Yes	Yes
Method of randomization described?	N.a.	No	Yes	Yes	N.a.	N.a.	No	Yes	N.a.	Yes	Yes
Method appropriate?	N.a.	No	Yes	Yes	N.a.	N.a.	No	Yes	N.a.	Yes	Yes
Rating	Weak	Strong	Strong	Strong	Moderate	Moderate	Strong	Strong	Moderate	Strong	Strong
C. Confounders											
Pre-intervention differences?	Can’t tell	No	No	Yes	Yes	Yes	Yes	Yes	No	Yes	Yes
Percentage confounders controlled for	Can’t tell	N.a.	N.a.	80–100% (most)	Can’t tell	80–100%	60–79% (some)	80–100% (most)	80–100%	80–100%	< 60% (few or none)
Rating	Weak	Strong	Strong	Strong	Weak	Strong	Moderate	Strong	Strong	Strong	Weak
D. Blinding											
Outcome assessors were blinded?	Can’t tell	Can’t tell	Can’t tell	Can’t tell	Can’t tell	No	Can’t tell	Yes	Can’t tell	No	Yes
Participants were blinded?	Can’t tell	Can’t tell	Can’t tell	Can’t tell	Can’t tell	Can’t tell	Yes	Can’t tell	Can’t tell	Can’t tell	Yes
Rating	Weak	Weak	Weak	Weak	Weak	Weak	Moderate	Moderate	Weak	Weak	Strong
E. Data collection methods											
Valid measures?	Can’t tell	Yes	Yes	Yes	Can’t tell	Yes	Yes	Yes	Yes	Yes	Yes
Reliable measures?	Can’t tell	Yes	Yes	Yes	Can’t tell	Can’t tell	Yes	Yes	Yes	Yes	Yes
Rating	Weak	Strong	Strong	Strong	Weak	Moderate	Strong	Strong	Strong	Strong	Strong
F. Withdrawals and drop-outs											
Numbers and reasons reported per group?	Yes	Can’t tell	No	Yes	Can’t tell	No	No	No	Yes	Yes	No
Percentage completing study?	N.a.	80–100%	60–79%	80–100%	Can’t tell	80–100%	60–79%	< 60%	80–100%	80–100%	< 60%
Rating	N.a.	Strong	Moderate	Strong	Weak	Weak	Weak	Weak	Strong	Strong	Weak
Total A-F:	WEAK	WEAK	WEAK	WEAK	WEAK	WEAK	MODERATE	MODERATE	WEAK	WEAK	WEAK
Number of ‘strong’ ratings	0/6	4/6	3/6	4/6	0/6	1/6	2/6	3/6	3/6	4/6	3/6
G. Intervention integrity											
Percentage participants received intervention?	Can’t tell	80–100%	60–79%	80–100%	80–100%	80–100%	60–79%	80–100%	Can’t tell	80–100%	60–79%
Intervention consistency measured?	Can’t tell	Yes	Yes	Yes	Yes	No	Yes	Yes	Can’t tell	Can’t tell	Yes
Confounding unintended intervention?	Can’t tell	Can’t tell	Can’t tell	Can’t tell	Can’t tell	Can’t tell	Can’t tell	Can’t tell	Can’t tell	Can’t tell	Can’t tell
H. Analyses											
Unit of allocation	Individual	Individual	Individual	Individual (family)	Individual	Individual	Individual	Individual (family)	Individual	Individual	Individual
Unit of analysis	Individual	Individual	Individual	Individual	Individual	Individual	Individual	Individual	Individual	Individual	Individual
Appropriate statistical methods?	Yes	Yes	Yes	Yes	Yes	Yes	Yes	Yes	Yes	Yes	Yes
Analysis by intention-to-treat status	No	Can’t tell	No	Yes	No	Can’t tell	Can’t tell	No	Can’t tell	No	Can’t tell

## Study findings

### Grief outcomes

One study comparing an intervention with a passive control group provided some evidence of positive effects on grief outcomes. An 8-week support group program facilitated by a mental health professional and a trained volunteer found a greater decrease in grief feelings in the intervention group than in the control group [36].

Six studies with an active control group or a treatment-as-usual condition reported mixed findings. De Groot et al. [41] found no differences between a 4-session family-based psychotherapy and treatment-as-usual on measures of complicated grief, although there was a trend towards reduced maladaptive grief reaction and perceptions of being to blame for the death. A secondary analysis of the same sample comparing participants with suicidal ideation with those who reported no ideation found a non-significant decrease in complicated grief in the former group [42]. Wittouck et al. [44] assessed the effectiveness of an intervention based on cognitive-behavioral therapy (CBT) and psycho-education, using complicated grief as the outcome. Although grief reduced in the intervention group, no differences were found on the development of complicated grief eight months after the intervention. Zisook et al. [40] compared the effectiveness of antidepressant medication alone or in combination with complicated grief therapy for different groups of bereaved (bereaved by suicide, by accident/homicide, by natural causes). Complicated grief therapy resulted in similar reduction of complicated grief symptoms in the three bereaved samples, though the sample sizes may have been too small for detecting statistically significant differences [40].

A study comparing effects of a professionally led group psychotherapy and a social group program for widows bereaved through suicide [34, 35] found that grief symptoms reduced in the therapy group [34], although a repetition of the study with a larger sample failed to replicate this effect [35]. A study comparing the effects of a death-related writing task intervention with a control condition involving trivial writing tasks yielded mixed findings [37]. Both groups experienced a significant reduction in grief levels, but the suicide-related grief aspects were more reduced in the intervention group than in the control group [37].

### Psychosocial outcomes

Three studies with passive control groups focused on psychosocial outcomes, such as depression, anxiety, self-efficacy, social acceptance, and alcohol and drug use, in young people bereaved through suicide. Pfeffer et al. [38] demonstrated that a 10-week psychologist-facilitated group therapy program for children reduced anxiety and depression but not post-traumatic stress of social adjustment at 12-weeks follow-up. A psycho-educational component for parents may have contributed to the positive effects. It is worth noting, however, that there was significant attrition in the control group and follow-up beyond 12 weeks was not included. Sandor et al. [39] reported the effects of a series of three church-based support meetings following a suicide in the community. Modest positive effects were found in the intervention group in terms of greater self-efficacy, social acceptance and job competency, up to two months after the intervention. No effect on internalization, externalization, depression, risk behavior or drug and alcohol consumption was found in the only school-based intervention included in the current review [43].

Studies assessing the effects of an intervention for adults against an active control group found little evidence of effectiveness regarding psychosocial outcomes, including depression, distress, problem solving, and social adjustment. Participants in a weekly, 4-month, support group program with an educational component reported less painful emotions, and more positive emotions, insights, and problem-solving skills compared to non-help-seeking people bereaved by suicide and help-seeking individuals in psychotherapy [33]. However, a suicide and an attempted suicide occurred in the intervention group, and the study quality is unclear. The above-mentioned study by Constantino and Bricker [34] also looked at depression, distress and social adjustment and found significant reduction in depression and distress in both groups, whereas social adjustment improved in the social group only [34]. However, a repetition of the study with a larger sample did not find any difference between the two groups [35]. A study assessing the effects of an intervention based on CBT and psycho-education on complicated grief found reduced depression and passive coping styles in the intervention group, although no differences were found regarding depression at eight-month follow-up [44].

### Suicide-related outcomes

No study reported on non-fatal or fatal suicidal behaviour, still, three previously mentioned studies reported on suicidal ideation as an intervention outcome. Wittouck et al. [44] found no statistically significant differences between study groups regarding suicidal ideation either immediately after completion of therapy or eight months post intervention. De Groot et al. [41] found no differences between family-based psychotherapy and treatment-as-usual on measures of suicidal ideation. However, Zisook et al. [40] found that complicated grief therapy resulted in a significant reduction in suicidal ideation in participants bereaved through suicide.

## Discussion

This systematic review aimed to establish the evidence for the effectiveness of suicide bereavement interventions. Unlike previous reviews, it only included controlled studies, and reported on grief, psychosocial and suicide-related outcomes. Despite a substantial increase in suicide bereavement research over the last decades [29, 30] the search identified only 12 papers, reporting on 11 studies, published over 35 years (1984–2018). Almost three quarters of the studies (8 out of 11 studies) were conducted in the USA, and the remaining three in Western Europe. It is not clear whether their results would be replicated in other cultural settings and (mental) health care systems [53]. None of the studies particularly addressed interventions for people aged 65+, although there is some evidence that elderly suicide survivors may experience unique challenges while coping with their loss [54]. Most intervention studies included more female participants than male participants. As there are gender differences in the experience of suicide loss and coping strategies between females and males [55], the unintended focus on effectiveness of interventions in female-dominated samples [56] creates a significant gap in the literature and postvention practice.

Studies identified in this review examined a wide range of outcomes related to grief, psychosocial functioning, and suicidal ideation. Given the diversity of outcomes across studies, the wide range of measures employed, and the methodological limitations, at this stage it is not possible to univocally indicate effective interventions targeting issues related to bereavement through suicide. Two studies that tested CBT-based interventions targeting complicated grief, such as an intervention with psycho-education [44] and targeted complicated grief therapy [40], yielded some positive short-term results. Nonetheless, De Groot et al. [41] in a study of a family CBT grief counseling program did not report lower levels of complicated grief. Similarly, other intervention studies reporting on grief reactions in general indicated some inconsistent positive results regarding broadly defined self-reported grief feelings [36], grief symptoms measured by the Grief Experience Inventory [34, 35, 47], and the Grief Recovery Questions [37, 57]. This lack of evidence regarding effective grief interventions (as well as evidence of lack of effectiveness) for suicide survivors is concerning given their susceptibility to complicated grief reactions [10], and more suicide-specific reactions, such as feelings of rejection and struggles with ‘why’-questions [7–9].

Despite research findings indicating increased risk of suicidal ideation and behaviour among people bereaved through suicide [8, 14, 16], only three studies identified in the review looked at a suicide-related outcome, which was suicidal ideation. Of interest, these three studies were relatively recent RCTs and evaluated effectiveness of a CBT-based psychotherapy intervention [40, 41, 44]. Again, results were mixed, as only Zisook et al. [40] reported a significant reduction in suicidal ideation in both the suicide bereaved and the non-suicide bereaved groups. None of the reviewed studies looked at suicidal behaviour as an outcome. This may be related to relatively short intervention follow-up periods, statistical rarity of non-fatal and fatal suicidal behavior among the bereaved by suicide [16], and relatively small sample sizes [7, 8].

All reviewed studies reported on effectiveness of interventions in terms of various psychosocial outcomes, such as depression and/or anxiety [e.g., 34, 38], posttraumatic stress [38], distress and social adjustment [34], self-efficacy [39], problem solving [33], and substance use [43]. Particular interventions were related to particular positive outcomes, e.g., a group therapy for children [38], CBT-based psycho-educational intervention [44], and group psychotherapy for widows [34], found significant reductions in depression. Nonetheless, the diversity of intervention settings, populations and measures used, along with very limited replicability of effectiveness studies, limit conclusions and implications for postvention practice. Again, this is concerning in the light of possible negative grief, mental health, and suicide-related sequelae of suicide loss [8, 16] and support needs of the bereaved [21].

### Factors affecting effectiveness of interventions

Of the interventions with a passive control group, effective interventions [36, 38] were delivered over time (eight and 10 weeks respectively). Both interventions were provided by trained facilitators (trained volunteer and clinician, and clinicians only, respectively), and included supportive, therapeutic and educational aspects. The use of manuals or guidelines may help guiding the intervention [37, 38, 40–42]. Involving parents [38], or the wider community [39] may contribute to the effectiveness. A common factor in the effective studies comparing different interventions (involving an active control group) is the finding that grief-specific interventions may yield stronger effects on grief outcomes compared to interventions targeting other outcomes [34, 37], though other studies failed to find such an effect [35]. The ineffective interventions with a passive comparator [43, 44] comprised shorter interventions (one and four sessions, respectively), and focused on complicated grief [44]. Other RCTs [40, 41] also failed to find positive effects on complicated grief outcomes. In addition, psychosocial characteristics of the bereaved who enter psychotherapy or other grief interventions may impact effectiveness of an intervention. For instance, De Groot et al. [42] found that participants who reported suicidal ideation benefited more from a family-based psychotherapy intervention in terms of both reduced risk of maladaptive grief reactions and lowered suicidality, than participants without suicidal ideation.

### Limitations

Despite extensive systematic searches the review included 12 papers reporting on 11 studies only. Overall, the quality of the studies is weak, especially with regard to selection bias, blinding, and withdrawals and dropouts. Assessing the quality of studies published before 1998 was particularly difficult as the articles did not provide information required to address quality criteria listed in the Quality Assessment Tool for Quantitative Studies [32] used in this review. Also, some of the newer studies did not report on all the quality criteria. In both scenarios, this resulted in ratings of ‘weak’ on those components, and it is possible that the overall study quality was higher than reported in the publications. This observation points out that the quality of the reporting of the intervention studies remains a concern.

### Implications

Due to the limited number and relatively modest quality of the studies the implications for practice are not robust. This finding is particularly concerning from the point of view of clinical and community postvention practice. Despite five decades of research there is still a lack of evidence as to which interventions are effective for suicide bereavement and its associated outcomes, including complicated grief. It also remains unclear which intervention modalities delivered in particular settings, such as schools [43], participants’ own homes [41, 44] or clinical settings [40], are most helpful for suicide survivors across a range of age, gender and/or national/cultural groups. Initiatives, such as the Core Outcome Measures in Effectiveness Trials [58] and the International Consortium for Health Outcomes Measurement [59], support development of an agreed set of standardized outcome measures or “core outcome sets”, for particular (mental) health conditions. Development of a “core outcome set” for suicide bereavement interventions could facilitate collection and reporting of comparable effectiveness data, thus addressing heterogeneity of outcomes and measures reported in this systematic review.

Still, the review identified crucial information for service providers and bereaved individuals seeking support. Suicide grief interventions need to include a sufficient number of sessions over a sufficient length in time. Interventions should include supportive, therapeutic and educational aspects, and must be led by trained facilitators, who may benefit from the usage of manuals. Involving the social environment of the bereaved individuals may contribute to the effectiveness. Future research should focus on grief and complicated grief interventions of sufficient duration. Selection procedures, sample sizes, randomization and blinding need specific attention. Appropriate measures of grief, complicated grief and mental health should be applied. Both short-term (e.g., post-intervention) and long-term follow-up (e.g., several months) should be assessed. Also, echoing a recommendation formulated a decade ago [24], qualitative research, currently lacking, may increase our understanding of how the bereaved experience the interventions, and what they find helpful or not. Future research should pay equal attention to males and females, and to interventions in different age groups, especially with regard to bereaved older adults, an age group currently overlooked.

## Conclusions

This systematic review found scant evidence of effectiveness of suicide grief interventions. Whereas there is some evidence of effectiveness of general suicide grief interventions, evidence of the effectiveness of complicated grief interventions after suicide is lacking. There is a clear need for the methodologically sound conducting and reporting of controlled studies across the life-span. Further research is essential to prevent adverse grief, mental health ramifications and suicidal behaviour in people bereaved by suicide.

## Abbreviations

CBT: Cognitive-behaviour therapy
MeSH: Medical subject headings
PRISMA: Preferred reporting items for systematic reviews and meta-analyses
PTSD: Post-traumatic stress disorder
RCT: Randomized controlled trial
